# Immunohistochemical Study of Glucose Transporter GLUT-5 in Duodenal Epithelium in Norm and in T-2 Mycotoxicosis

**DOI:** 10.3390/foods9070849

**Published:** 2020-06-29

**Authors:** Piret Hussar, Florina Popovska-Percinic, Katerina Blagoevska, Tõnu Järveots, Ilmārs Dūrītis

**Affiliations:** 1 Faculty of Medicine, University of Tartu, Ravila 19, 50411 Tartu, Estonia; 2 Faculty of Veterinary Medicine, Ss.Cyril & Methodius University in Skopje, 1000 Skopje, North Macedonia; popovskaf@gmail.com; 3 Laboratory for Molecular Food Analyses and Genetically Modified Organism, Food Institute, Faculty of Veterinary Medicine, 1000 Skopje, North Macedonia; katerinab@fvm.ukim.edu.mk; 4 Institute of Veterinary Medicine and Animal Sciences, Estonian University of Life Sciences, 51006 Tartu, Estonia; tonu.jarveots@emu.ee; 5 Faculty of Veterinary Medicine, Latvian University of Agriculture, LV 3004 Jelgava, Latvia; ilmars.duritis@gmail.com

**Keywords:** GLUT-5, duodenal epithelium, T-2 mycotoxicosis

## Abstract

Although patterns of glucose transporter expression and notes about diseases leading to adaptive changes in intestinal fructose transport have been well-characterized, the connection between infection and fructose transportation has been lightly investigated. Up to now only few studies on GLUT-5 expression and function under pathological conditions in bird intestines have been carried out. The aim of our current research was to immunolocalize GLUT-5 in chicken duodenal epithelium in norm and during T-2 mycotoxicosis. Material from chicken *(Gallus gallus domesticus)* duodenum was collected from twelve seven-day-old female broilers, divided into control group and broilers with T-2 mycotoxicosis. The material was fixed with 10% formalin and thereafter embedded into paraffin; slices 7 μm in thickness were cut, followed by immunohistochemical staining, according to the manufacturers guidelines (IHC kit, Abcam, UK) using polyclonal primary antibody Rabbit anti-GLUT-5. Our study revealed the strong expression of GLUT-5 in the apical parts of the duodenal epithelial cells in the control group chickens and weak staining for GLUT-5 in the intestinal epithelium in the T-2 mycotoxicosis group. Our results confirmed decreased the expression of GLUT-5 in the duodenal epithelium during T-2 mycotoxicosis.

## 1. Introduction

Glucose, being a main energy source [1], and glucose transporters being present in all phyla, the proteins belonging to the GLUT or SLC2A family are found in most mammalian cells. The main role in transmembrane monosaccharide transport belongs to the glucose transporter (GLUT) proteins, which influence blood sugar regulation [2,3]. Glucose reaches into the target cells, passing through the intestinal epithelium. Glucose is absorbed by two structurally and functionally different groups, exhibiting different substrate specificities, kinetic properties and tissue expression profiles: active—absorption mediated by sodium-dependent glucose transporters (SGLTs); diffusive—glucose transporter facilitators (GLUTs) [4,5]. The GLUT family can be divided into three subclasses on the basis of sequence similarities: Class I—glucose transporters; Class II—formed by fructose transporters; Class III—belong structurally atypical transporters [6].

The knowledge of glucose transporters in avians is fragmented. It is known that diets of many birds change with seasons, causing the carbohydrate levels to vary accordingly. Comparing the dietary regulations of intestinal glucose transport with other species (fishes, amphibians, mammals) the transport in birds does not change, which may be caused by the predominance of passive glucose transport [7]. It is also known that birds maintain higher plasma glucose concentrations than other vertebrates of similar body mass and are mostly insensitive to the regulation of plasma glucose concentrations by insulin [4]. Glucose is absorbed by the avian gastrointestinal tract by sodium-glucose transporters and glucose transport proteins. In the small intestine, four of the most abundant transporters are found: GLUT-1, GLUT-2, GLUT-5 and SGLUT1 [8], of which GLUT-5 is a high affinity fructose transporter. The main site for GLUT-5 absorption is the epithelium of jejunum and in the last part of ileum [9,10], as the epithelial cells absorb hexoses from the intestinal lumen and export sugars into blood. In the beginning, absorption depends on active transport in the apical brush border membrane, after which the hexoses move out of the enterocytes by passive transport [11].

Glucose transporter-5 (GLUT-5), the main apical fructose transporter and the transporter which is specific only for fructose, allows fructose, the sweetest of all natural sugars, to be transported from the intestinal lumen into the enterocyte by facilitated diffusion, due to fructose’s high concentration in the intestinal lumen [12]. According to the literature, there are studies which support the direct relationship between increases in the consumption of fructose and the increase in the diabetes type-2 [13]. According to previous studies, the small intestine regulates fructose absorption from dietary sources and expresses the greatest amount of GLUT-5 in mammals [14].

Although it is known that infectious diseases lead to changes in intestinal absorptive function, the connection of infection and fructose transport has been slightly studied. Up to now, only few studies on intestinal GLUT-5 expression and function during diseases have been carried out [15,16].

T-2 mycotoxin, the basic type A trichothecene mycotoxin, has been regarded to be the most toxic trichothecene. In dynamic cell proliferation tissues, such as the gastrointestinal tissues, T-2 mycotoxin poses different toxic effects [17,18]. It was shown that, in poultry, the T-2 toxin elicits genetic, cellular toxic and immunomodulatory effects, influencing the cells of the digestive, nervous and integumentary system, as well as on the impairment of poultry performance [19]. Symptoms of T-2 mycotoxicosis in chicken manifest in growth retardation, lower feed intake, reduced egg production with thinner eggshells, impaired egg hatch, leucopenia and the cyanosis of the comb. Besides, T-2 mycotoxin is among to the most essential trichothecene mycotoxins, which occurs in several agricultural products [20], causing severe diseases among humans and animals, which can even lead to death [21]. Animals are exposed through food to T-2 mycotoxins, whose initial interaction is with the gut epithelium.

As animals are exposed through food to T-2 mycotoxins, one of the most deadly toxins of the trichothecene group and, due to the absence of data about the effect of mycotoxicosis on GLUT-5 in the intestines of birds during their first post-hatching week, the aim of our current research was to immunolocalize GLUT-5 in the seven-day old chicken duodenal epithelium in norm and during T-2 mycotoxicosis.

## 2. Materials and Methods

The duodenal material was collected from twelve seven-day-old female *Ross* broilers *(Gallus gallus domesticus),* obtained from a commercial Macedonian hatchery, divided equally into control and T-2 mycotoxin groups—six broilers in each group. To evoke mycotoxicosis, the Fusarium toxin, trichothecene T-2 (Sigma, Steinheim, Germany) was applied from the fourth day of the experiment per os for three consecutive days to the T-2 mycotoxin group with a syringe in a dosage 0.250 mg/day/bird. To the birds of both groups, feed and water were given ad libitum. Then, 24 h after the last dosage of the T-2 mycotoxin, the chickens were sacrificed by intracardiac overdose of 0.5 mL 20% sodium pentobarbital, and material from the middle part of the duodenum was removed. Specimens 0.5–1.0 cm in diameter were fixed with 10% buffered formalin and embedded into paraffin. Thereafter the slices, 7 μm in thickness, were cut (microtome Leica 2135), floated on Poly-L-Lysine coated slides (O. Kindler GmbH, Freiburg, Germany), deparaffinized with xylene and rehydrated in a graded series of ethanol, followed by the methods of routine histology and immunohistochemistry. For routine histology, the slices of the tissues were stained by the Hematoxylin and Eosin method, according to the standardized tissue histological procedure [22].

For immunohistochemistry, endogenous peroxidase activity was blocked with 3% H2O2. For staining, the sections Immunohistochemistry kit (IHC kit, Abcam, Cambridge, UK) was used, according to the manufacturer’s guidelines. Polyclonal rabbit antibody GLUT-5 served as the primary antibody (Abcam, UK). The sections were pre-treated by heat mediated antigen retrieval using 0.01 mol/L sodium citrate buffer (pH 6; 20 min), thereafter incubated with primary rabbit polyclonal antibody to glucose transporter-5 (GLUT-5) (Abcam, UK) in 1/400 dilution for 30 min at 37 °C. Biotinylated secondary antibody for 1 h at room temperature and streptavidin-conjugated peroxidase were used for detection where 3.3′-diaminobenzidine tetrahydrocloride (DAB) served as chromogen. Negative controls were processed as above, but did not contain primary antibodies. As positive controls, human intestine sections for GLUT-5 are available for comparison on the Abcam antibody producer homepage (http://www.abcam.com) as examples for the antibody immunohistochemistry on paraffin-embedded tissues (IHC-P).

Photos of the slides were taken with the Zeiss Axioplan-2 Imaging microscope (Karl Zeiss, Göttingen, Germany), equipped with a digital camera (AxioCam HRc, Göttingen, Germany) and connected to the computer. The photos were saved to the computer and analyzed by visual control by three independent researchers.

The Ethical Committee of Ss. Cyril and Methodius University in Skopje, in conformity with the recommendation provided in the European Convention for the Protection of Vertebrate Animals used for Experimental and Other Scientific Purposes (ETS no.123, Approval No. 03-7534, 12.04.2013), approved the husbandry and experimental procedures of the study.

## 3. Results

### 3.1. Light Microscopy

**Figure 1:**
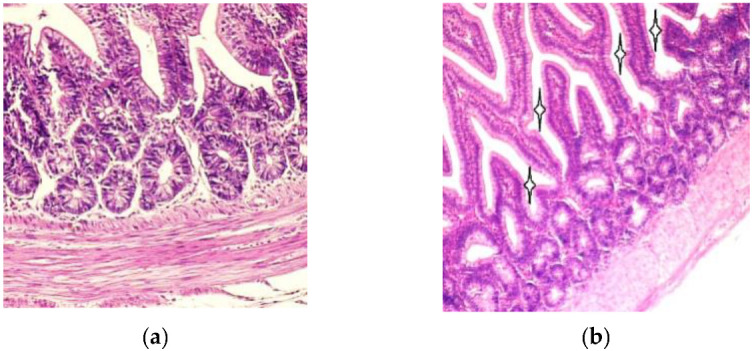
(**a**) Duodenal villi and crypts in normal seven-day-old chicken mucosa, Hematoxylin–eosin 200×; (**b**) Villi and cryptae duodenales in toxicated seven-day-old chicken mucous layer. Note the narrow duodenal villi and enlarged intervillar spaces (asterisks). Hematoxylin–eosin, 100×.

For overall histological assessment the sections of duodenum were examined by light microscopy. The duodenal villi were lined by simple columnar epithelium. The basal parts of the intestinal villi were wider than the apical parts, as shown in Figure 1a. Compared to the T-2 mycotoxin group, the intestinal villi were slightly larger and the intervillar spaces narrower in the un-toxicated birds than in birds of the T-2 mycotoxin group, as shown in Figure 1b.

### 3.2. Immunohistochemistry

**Figure 2:**
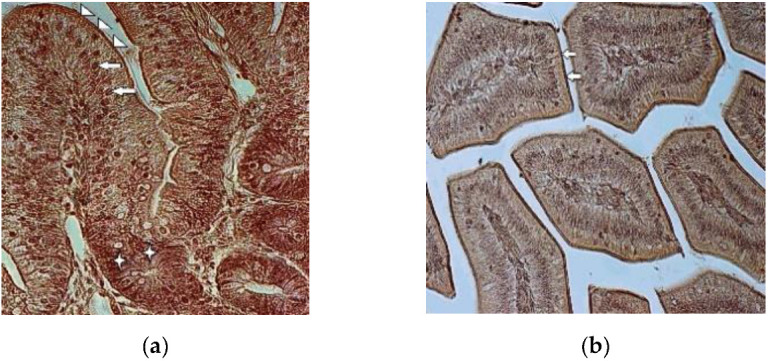
**(a)** Expression of GLUT-5 in seven-day-old broiler’s duodenal epithelium (control group). Strong staining for GLUT-5 in brush border membrane (arrowheads) and nuclei of enterocytes (arrows). Note the strongly stained cells in the intestinal crypts (asterisks) 400×; (**b**) Expression of GLUT-5 in seven-day-old toxicated broiler’s duodenal epithelium. The cytoplasm and nuclei of enterocytes are stained weakly. Note the strongly stained brush border membranes (arrows) 200×.

In the control group, the strong staining of the chicken duodenal epithelium was noted in the brush border membranes of intestinal villi, enterocyte’s nuclei and in the cytoplasm of the cryptal cells, as shown in Figure 2a. The expression of GLUT-5 was moderate in the cytoplasm of the enterocytes in the duodenal villi. The goblet cells remained mainly unstained.

Compared to the control group chickens, weak staining for GLUT-5 was noted in the duodenal epithelial cells in the T-2 mycotoxicosis group. Both cell types—enterocytes and goblet cells—were stained weakly. Stronger staining was noted only in the brush border membranes of the intestinal villi, as shown in Figure 2b.

## 4. Discussion

The gastrointestinal organs are among the first organs coming into contact with mycotoxins of dietary origin. The small intestine begins with the duodenum where the absorption of nutrients starts and it receives partially digested food directly from the stomach. It consists of the typical three layers—mucous, muscular and serous layers—common to all of the hollow organs of the gastrointestinal system. Our overall histological assessment of the intestinal mucosal tunic, found in both the control group and the birds with mycotoxicosis, is in line with data from other authors regarding the existence of a simple columnar epithelium lining with goblet cells and enterocytes (columnar cells), and the presence of villi with wider basal and narrower apical parts [23,24,25]. Our investigations revealed narrower duodenal villi and larger crypts in the T-2 mycotoxicosis group, compared to the control group. These findings are in accordance with the data about the effect of alfatoxin B1 on broiler chicken duodenum, where the decreased villus area and villus height in the duodenum was shown after the administration of the alfatoxin B1 [26,27]. The authors suggested a compensation for the reduced surface area of the duodenum villi resulting from reduced villi heights in these birds.

In the gastrointestinal system, the glucose transporter expression is the greatest in the small intestine, where the absorption for monosaccharides depends on the sodium-dependent glucose transporter SGLUT1 and the facilitated-diffusion glucose transporters located in the intestinal epithelium [28,29]. Identical expressions of GLUT2 and GLUT5 mRNA have been noticed from the proximal to middle parts of the small intestine [29,30] where GLUT-5 is located on the apical membrane of epithelial cells. While galactose and glucose transport is mediated by SGLT1 [7,31,32], GLUT-5 only mediates the uptake of fructose [33,34,35], whose activity changes during pathological conditions. Generally, it was found that the activity and expression of GLUT-5 was reduced during inflammation and sepsis in rabbits [36,37]. According to those authors lipopolysaccharide and tumor necrosis factor-α, as the main causes of sepsis, provoked decreased fructose absorption in the jejunum. In humans it has been noted that the infection caused by *Helicobacter pylori* also reduces the expression of intestinal GLUT5 [16]. Decreased expression of GLUT-5 in the duodenal epithelial cells in the T-2 mycotoxicosis group found in our study points towards reduced fructose transportation in the diseased gut epithelium.

T-2 mycotoxin is a naturally occurring mold byproduct of *Fusarium spp*. fungus, toxic both to humans and animals. The ingestion of T-2 mycotoxin may occur because of the intake of moldy grains—barley, maize, rice, wheat, etc. The T-2 mycotoxin is specific because the systemic toxicity can result from different route of exposure—respiratory, oral and dermal [38]. The T-2 toxin can be absorbed through human skin, unlike most biological toxins [39]. Besides skin, it causes symptoms related to respiratory and gastrointestinal organs. The fact that it is delivered by water, droplets, aerosols from various dispersal systems and food also makes it a potential biological weapon [40]. In vivo and in vitro T-2 mycotoxin can inhibit DNA and RNA, as well as inhibit protein synthesis [41]. These effects led to apoptosis in various tissues, including immune- and gastrointestinal systems [42]. In immune systems, it inhibits erythropoiesis in the bone marrow and spleen by disturbing the antibody production [43]. According to earlier research, T-2 mycotoxin is able to inhibit IL-2 and IL-5 production by T cells. It has been reported that low concentrations of T-2 mycotoxin ingestion changes Toll-like receptor activation, interfering with the initiation of the inflammatory immune responses against viruses and bacteria. Thus, the mycotoxins may increase the receptivity of animals and humans to infectious diseases [44,45]. Mycotoxins elicit similar toxic effects among humans and animals. In bird intestines, various methods have been used to study the effects of toxins [46,47]. Some data revealed the immuno- and cytotoxic effect of ochratoxin A on intestinal epithelium and MALT-system (mucosa-associated lymphoid tissue), modifying the intestinal barrier and thus increasing receptiveness to different associated diseases [41]. A decreased glucose uptake was registered after the oral administration of the T-2 toxin [48].

In our study, GLUT-5 immunolocalized in duodenal mocosa revealed weak expression in the duodenal epithelial cells in one-week-old T-2 toxicated broilers, compared to the control group after only three days of T-2 mycotoxin administration. As the gastrointestinal system is exposed to all the mycotoxins in contaminated feed, and GLUT-5 expression levels are significantly affected by various diseases and metabolic disorders, such as diabetes, hypertension, obesity, inflammation and carcinogenesis [14,35,49], more morphometrical and functional studies on hexose transporter expression in norm and during diseases are required in the future.
